# Annotated universal dependencies dataset for literary and educational Uzbek texts

**DOI:** 10.1016/j.dib.2026.112857

**Published:** 2026-05-19

**Authors:** Sanatbek Matlatipov, Mersaid Aripov, Makhmud Bobokandov, Gayrat Matlatipov

**Affiliations:** a The National University of Uzbekistan is named after Mirzo Ulugbek. Universitet Street, 4, Olmazor District 100174, Tashkent City, Uzbekistan; b Urgench State University named after Abu Rayhn Biruni, Khamid Alimdjan 14 220100, Urgench City, Uzbekistan

**Keywords:** Dependency treebank, Natural language processing, Turkic languages, Uzbek language, Syntax, Morphological features, Low-resource languages, Part-of-Speech Tagging

## Abstract

This data article describes an Uzbek Universal Dependencies (UD) treebank released as a manually curated gold-standard dataset. The resource contains 681 sentences (7542 tokens) drawn from literary and educational Uzbek texts, providing a domain-specific complement to previously available web-based or news-oriented materials. Annotation was carried out in the INCEpTION environment by a five-member team comprising three linguists and two NLP engineers. The workflow followed the UD v2 framework and included calibration-stage agreement assessment, full-corpus double annotation, and adjudication to improve annotation consistency. Agreement measured on the shared calibration material was high across lemmatization, universal part-of-speech annotation, and complete morphological feature–value bundles. The released dataset contains final adjudicated gold-standard annotations, including lemmas, UPOS tags, morphological features, and basic dependency relations in standard CoNLL-U format, and has been validated for compatibility with the Universal Dependencies ecosystem. As an openly reusable Uzbek syntactic resource, it can support the development and evaluation of POS taggers, morphological analyzers, and dependency parsers, while also enabling comparative and cross-lingual studies for low-resource languages.

Specifications Table

**Table:** 

Subject	Computer Sciences
Specific subject area	Natural Language Processing (NLP); Computational Linguistics; Uzbek language; Dependency Parsing for Low-Resource Agglutinative Languages.
Type of data	Table (CoNLL-U), Text (Raw sentences), Analyzed, Filtered, ProcessedCoNLL-U Format The dataset uses the CoNLL-U format, a standard for Universal Dependencies annotation [1]. Files are UTF-8 plain text where each sentence is separated by a blank line, and comment lines start with a hash (#). Each word token is represented by a single line containing 10 tab-separated fields: 1. ***ID*:** Word index (integer starting at 1). 2. ***FORM*:** The word form or punctuation. 3. ***LEMMA*:** The lemma or stem. 4. ***UPOS*:** Universal part-of-speech tag. 5. ***XPOS*:** Language-specific part-of-speech tag (underscore if unavailable). 6. ***FEATS*:** List of morphological features. 7. ***HEAD*:** ID of the current word's head (0 if root). 8. ***DEPREL*:** Universal dependency relation to the Head. 9. ***DEPS*:** Enhanced dependency graph (optional). 10. ***MISC*:** Any other annotation (e.g., spacing).
Data collection	The data was collected by manual annotation of sentences from selected Uzbek literary and educational sources: the fiction story "Maqar" [2], the book "Kun shundan boshlanadi" [3], and educational fairy-tale texts from ertak.uz. Written non-exclusive permission was obtained from the author and copyright holder of "Maqar" and "Kun shundan boshlanadi" to extract isolated sentences, annotate them linguistically, include them in UzUDT, and release the annotated sentence-level records as part of the public UD treebank. The annotation was performed in the INCEpTION platform by a team of linguists and NLP engineers using a gold-standard workflow that included calibration, double annotation, adjudication, and validation. The final dataset was checked with the official UD validation suite (*validate.py*) to ensure compliance with UD v2 standards. All sentences are in the Uzbek Latin script (the official script for modern Uzbek), and we avoided sentences containing non-standard or heavily dialectal forms. We targeted relatively self-contained sentences of moderate length (the average sentence length is ∼12 tokens), avoiding extremely long or overly simple sentences.
Data source location	Institution: National University of Uzbekistan named after Mirzo Ulugbek City: Tashkent Country: Uzbekistan
Data accessibility	Repository name: Universal Dependencies (GitHub/Official Website)Data identification number: ***uzudt***Direct URL to data: https://github.com/UniversalDependencies/UD_Uzbek-UzUDT/tree/master and https://universaldependencies.org/treebanks/uz_uzudt/index.htmlInstructions for accessing these data: The dataset is hosted as part of the official Universal Dependencies project (treebank: *uz_uzudt*). It is available under the standard open license provided by the UD framework; the sentence-level excerpts from “Maqar” and “Kun shundan boshlanadi” are included with written permission from the author and copyright holder.**Files:** • **uz_uzudt-ud-train.conllu**: This file contains the training split of the dataset. • **uz_uzudt-ud-test.conllu**: This file contains the testing split.
	• **stats.xml**: A machine-readable XML file which is statistical metadata about the treebank. • **eval.log**: The official Universal Dependencies validation and release-quality log for the treebank, documenting the PASSED status of the released CoNLL-U files, the Validity = 1 flag, and the UD release-quality rubric used to assign the treebank’s 2.5-star rating. • **README.md**: A documentation explains how the main tables and figures in this article relate to the released repository files, including corpus split statistics, validation logs, and statistics derived from stats.xml.
Related research article	*None*

## 1 Value of the Data

• UzUDT is a publicly available Universal Dependencies treebank for Uzbek that is fully manually annotated. The data provide high-quality syntactic and morphological annotation for 681 sentences (7542 tokens), covering lemmas, UPOS tags, morphological features, and dependency relations in a consistent CoNLL-U format. This fills a critical gap for Uzbek, which has been largely underrepresented in treebank-driven NLP resources.
• The corpus can be easily used for training and testing base Uzbek NLP tasks. Scholars can make use of the treebank to evaluate various projects such as part-of-speech taggers, morphological analyzers, dependency parsers, as well as carrying out studies on transfer learning in a multilingual setting within the UD scheme due to the existence of a train/test split.
• UzUDT supports typological and cross-lingual research on Turkic and low-resource languages. Because the treebank follows the UD v2 guidelines and uses the same annotation schema as other UD treebanks, it can be integrated into cross-lingual experiments, typological analyses, and comparative studies of agglutinative morphology and word order.
• A collection of auxiliary resources is also provided to describe the data’s internal structure and quality. Besides processing and analyzing all CoNLL-U-formatted resources, *stats.xml* offers detailed statistics for each corpus on POS tags, features, and dependencies, and *eval.log* contains scores from the UD evaluation and star ratings for each dataset.
• Cross-Lingual Compatibility: Researchers can reuse this dataset for cross-lingual studies and transfer learning. Because the annotations strictly follow the Universal Dependencies v2 guidelines, the data is directly compatible with treebanks from other Turkic languages (such as Turkish, Kazakh, and Uyghur). This facilitates comparative linguistic research and the development of multilingual NLP systems aimed at low-resource settings.

## 2 Background

Uzbek is a crucial Turkic language that has been largely understudied in terms of syntactically based natural language processing [4]. The main motivation for creating UzUDT was to establish a high-quality dependency treebank for Uzbek that would enable its integration into the Universal Dependencies ecosystem and downstream neural NLP pipelines such as Stanza [5]. The dataset was created under the Universal Dependencies v2 framework using the CoNLL-U [1] representation format. In contrast to existing web and news corpora [6], sentences were carefully selected from literary and educational sources and manually annotated for lemmas [7], universal part-of-speech categories [8], morphological features [9], and dependency relations [10]. At the same time, ongoing work on Uzbek sentiment analysis and aspect-based sentiment analysis [11,12] highlighted the lack of syntactic resources for more linguistically informed machine-learning models. Consequently, UzUDT was also designed as a reusable infrastructure for downstream Uzbek NLP tasks, including sentiment analysis and aspect-based sentiment analysis. In such tasks, dependency relations can link sentiment-bearing predicates or modifiers to their correct aspect targets and help identify the scope of negation, which is information that simple bag-of-words features cannot directly represent. The data article of interest documents the treebank itself, how it was annotated, and statistics on its validation, to allow other authors access to the same version of the data.

## 3 Data Description

The UzUDT treebank is distributed through the official Universal Dependencies repository under the name *UD_Uzbek-UzUDT*. The repository contains the following main files and folders:

• **uz_uzudt-ud-train.conllu:** Training portion of the treebank, consisting of 483 sentences and 5441 tokens.
• **uz_uzudt-ud-test.conllu:** Test portion, consisting of 198 sentences and 2101 tokens.
• **stats.xml:** An automatically generated XML file summarizing corpus statistics, including overall size, vocabulary, distribution of UPOS tags, morphological features, and dependency relations.
• **eval.log:** The official UD validation and release-quality log, documenting that the released CoNLL-U files passed validation, the release-validity flag is Validity = 1, and the UD release-quality rubric assigns the treebank a 2.5-star rating.
• **README.md:** A documentation file describing the treebank, its sources, intended use, and licensing conditions.
• License file and ancillary project files.

All annotated sentences are represented in the standard CoNLL-U format used for all corpora of the Universal Dependencies Project. Empty lines separate sentences, and lines beginning with # text = contain the source Uzbek sentence. Each non-empty line is associated with each token and is made up of ten fields separated by tabulation symbols: ID, FORM, LEMMA, UPOS, XPOS, FEATS, HEAD, DEPREL, DEPS, and MISC. **# sent_id = s207**

**# text = qirgʻoqdagi manzaralar odamni cheksiz zavqlantiradi.**

**Fig. 1:**
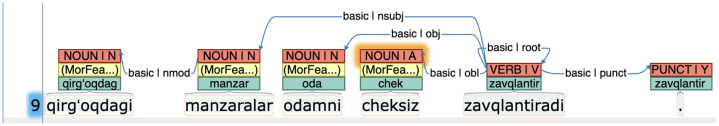
INCEpTION visualization of sentence s207, qirgʻoqdagi manzaralar odamni cheksiz zavqlantiradi (“The coastal sceneries delight the person infinitely”). The visualization displays token-level lemmas, POS tags, morphological features, and dependency arcs.

Table 1 presents one complete CoNLL-U record with each annotation field placed in a separate column, and Fig. 1 visualizes the same sentence in the INCEpTION interface, making the HEAD and DEPREL annotations easier to interpret.

**Table 1:** CoNLL-U annotation sample for sentence s207.

ID	FORM	LEMMA	UPOS	XPOS	FEATS	HEAD	DEPREL	DEPS	MISC
1	qirgʻoqdagi	qirgʻoq	NOUN	N	Case=Nom	2	nmod	_	_
2	manzaralar	manzara	NOUN	N	Case=Nom|Number=Plu	5	nsubj	_	_
3	odamni	odam	NOUN	N	Case=Acc	5	obj	_	_
4	cheksiz	chek	NOUN	A	Case=Nom	5	obl	_	_
5	zavqlantiradi	zavq	VERB	V	_	0	root	_	_
6	.	.	PUNCT	Y	_	5	punct	_	_

**# sent_id = s207**

**# text = qirgʻoqdagi manzaralar odamni cheksiz zavqlantiradi .**

Fig. 1 visualizes the same sentence shown in Table 1 in the INCEpTION interface. It highlights token-level annotation layers, including lemmas, UPOS/XPOS tags, morphological features, and dependency relations such as nmod, nsubj, obj, obl, root, and punct.

**Fig. 2:**
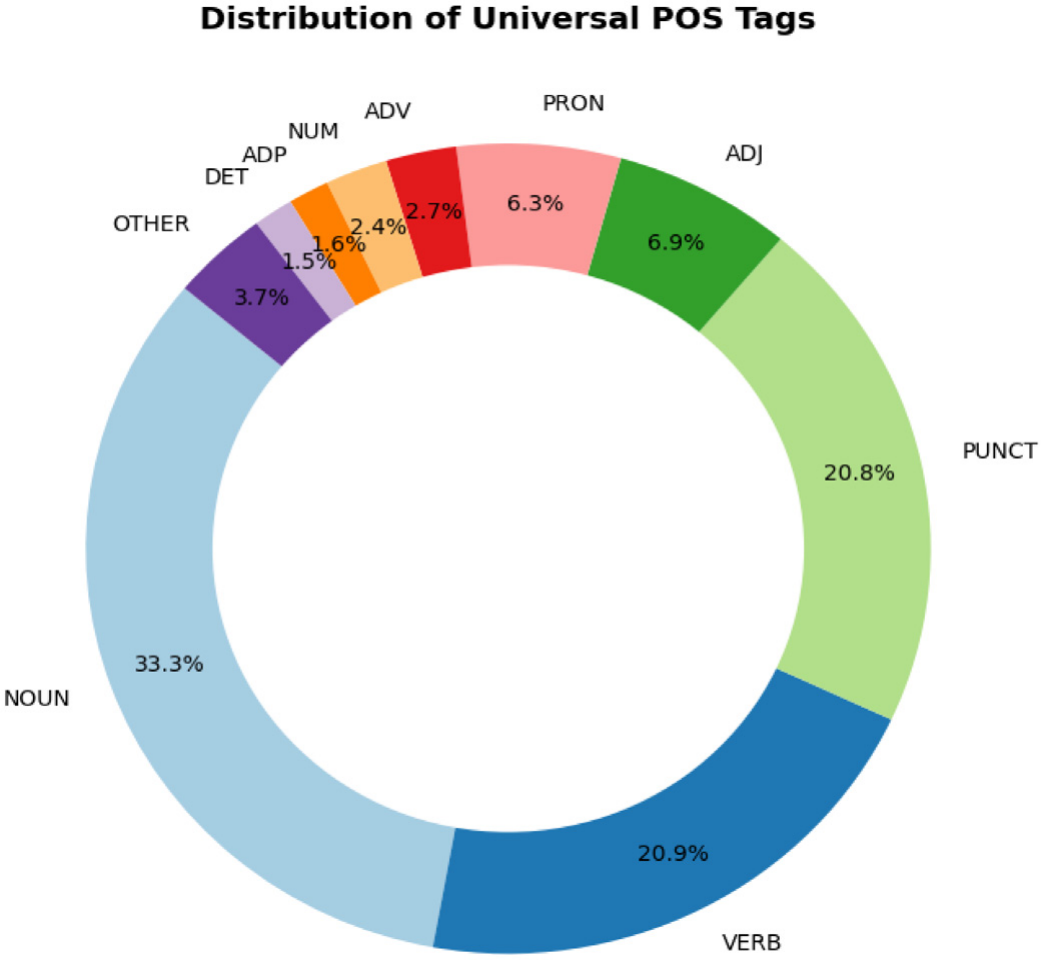
Distribution of Universal Part-of-Speech (UPOS) tags.

**Fig. 3:**
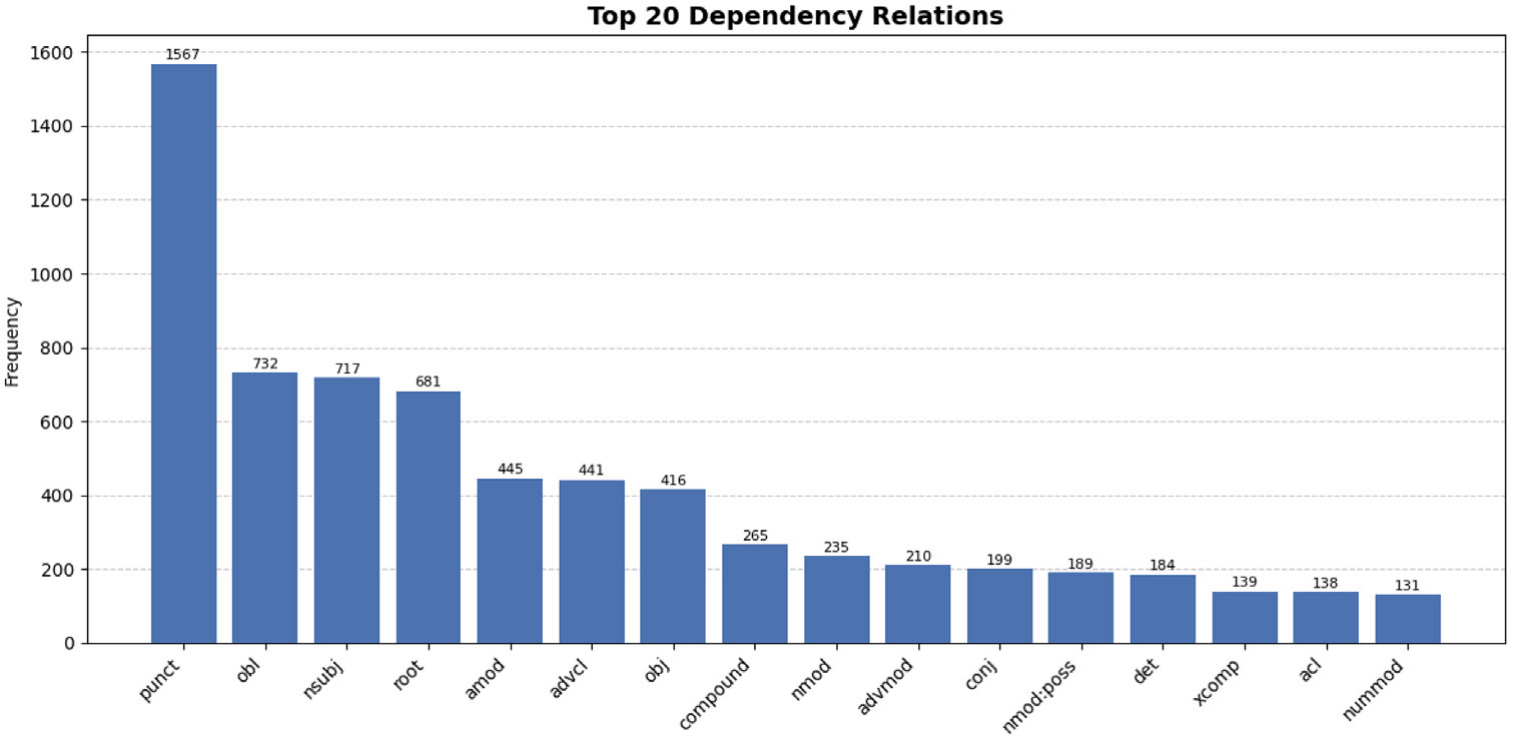
Frequency of the top 20 Universal Dependency relations.

At the token level, the released training split contains 5441 tokens and the test split contains 2101 tokens, giving 7542 tokens in total. As shown in Table 2, the treebank consists of 681 sentences distributed across two literary fiction sources and one educational fairy-tale source. The released corpus contains no multi-word tokens or fused forms. It covers 2560 lemmas and 3105 word forms, indicating moderate lexico-structural variety. The corpus attests all universal part-of-speech (UPOS) categories except SYM, which in UD is reserved for symbols such as mathematical operators, currency signs, and technical notation. SYM is absent because such symbols do not occur in the literary and instructional texts used to build the corpus. The UPOS distribution is visualized in Fig. 2, where NOUN (2508), VERB (1578), and PUNCT (1567) are the most frequent categories. Morphological annotation includes 54 feature–value pairs covering case, number, person, tense, aspect, mood, and voice; the most frequent feature–value pairs are summarized in Table 3, including Case=Nom, Number=Sing, and Person[psor]=3. The syntactic layer contains 38 dependency relation types, including general relations such as nsubj, obj, obl, and advcl, as well as relations relevant to Uzbek multiword predicates, such as compound:lvc and compound:redup. The most frequent dependency relations are visualized in Fig. 3, where punct, obl, nsubj, root, amod, advcl, and obj are among the highest-frequency relations.

**Table 2:** Per-source sentence distribution in the UzUDT train/test split.

Source	Domain/category	Train	Test	Total
Kun shundan boshlanadi	Literary, fiction	290	119	409
Maqar	Literary, fiction	145	59	204
ertak.uz fairy tales	Educational fairy tales	48	20	68
**Total**		**483**	**198**	**681**

**Table 3:** Frequency of the top morphological feature–value pairs.

Feature	Value	Count	Feature	Value	Count
Case	Nom	1537	VerbForm	Conv	324
Number	Sing	523	Mood	Ind	302
Person[psor]	3	433	Case	Acc	262
Person	3	412	Aspect	Perf	256
Number	Plur	384	PronType	Prs	239
VerbForm	Fin	382	Case	Dat	210
Tense	Past	361	Case	Gen	158
Number[psor]	Plur	353

Table 3 summarizes the most frequent morphological feature–value pairs in UzUDT, including the high-frequency values Case=Nom and Number=Sing.

## 4 Experimental Design, Materials and Methods

**Source Material.** The UzUDT corpus was constructed from narrative and educational domains to ensure syntactic diversity. Primary sources include the fiction stories "Maqar" [2] and "Kun shundan boshlanadi" [3], alongside publicly available educational fairy-tale texts from ertak.uz. Sentences were filtered for standard literary grammar and moderate length (avg. ∼12 tokens) to facilitate robust parser training.

**Fig. 4:**
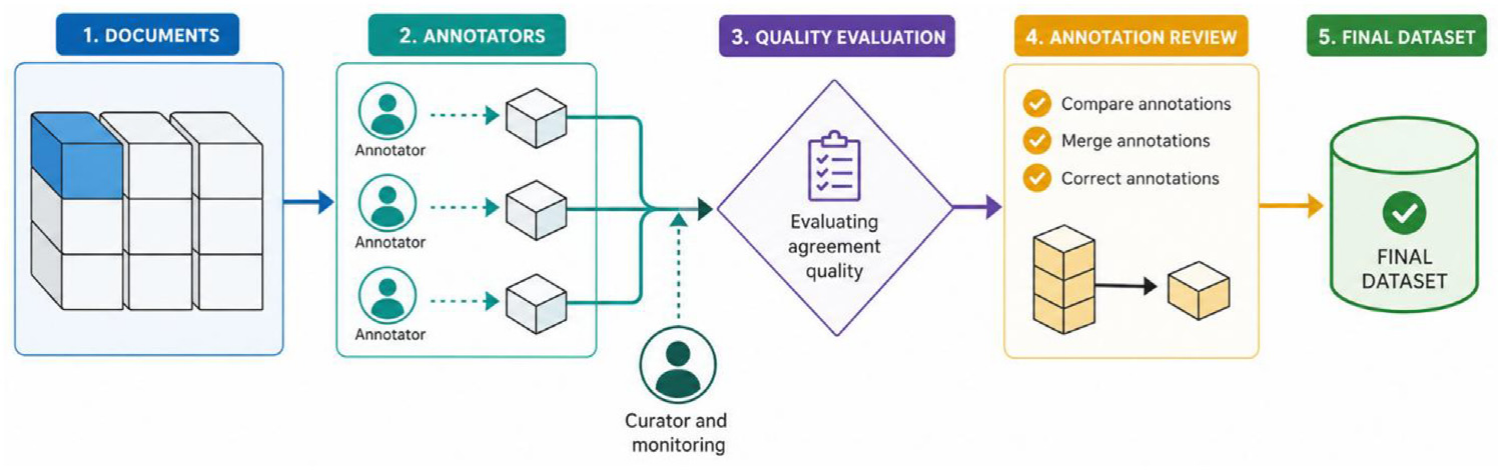
The manual annotation pipeline, illustrating the flow from document selection to final dataset generation via multi-stage annotation and curation.

**Annotation Workflow.** The annotation process was conducted using the INCEpTION platform [13]. As illustrated in Fig. 4, the annotation team consisted of five contributors: three linguist experts with native-level proficiency in Uzbek and two NLP engineers responsible for technical validation and guideline enforcement. The workflow followed a rigorous gold-standard methodology with three main stages:

1. **Calibration and guideline alignment:** The annotators first completed a shared calibration phase in INCEpTION to align their treatment of Uzbek-specific UD phenomena, including agglutinative case marking, possessor agreement, and null-copula constructions. Inter-annotator agreement was measured only on this shared calibration material, at the token level, for lemma annotation, UPOS tagging, and the complete set of morphological feature–value pairs assigned to each token.
2. **Full-corpus double annotation and adjudication:** The full corpus (681 sentences, 7542 tokens; 100% of the released dataset) was annotated in a double-annotation setting, with each sentence independently annotated by two annotators. Disagreements were identified using INCEpTION’s comparison view and resolved in regular adjudication meetings. A senior linguist served as the final arbiter in difficult cases to ensure consistency with the Universal Dependencies v2 guidelines. During adjudication, priority was given to guideline-conformant analyses, exact-match consistency for the complete set of morphological feature–value pairs assigned to each token, and uniform treatment of recurrent Uzbek-specific phenomena such as case marking, possessor agreement, and null-copula constructions.
3. **Inter-Annotator Agreement (IAA).** Agreement on the shared calibration material was high across all three annotation layers: lemma annotation (0.95), UPOS tagging (0.95), and the complete set of morphological feature–value pairs assigned to each token (0.90) [14]. These values were used as a calibration diagnostic before full-corpus annotation. A separate raw IAA score was not computed for the full 681-sentence corpus prior to adjudication; instead, full-corpus quality control was performed through double annotation, INCEpTION-based comparison, and adjudication. The pre-adjudication double-annotation files were treated as intermediate working artefacts, while the released files contain the final adjudicated gold-standard annotations.

***Technical Validation***. Following manual annotation and adjudication, the dataset was validated using the official Universal Dependencies validation suite (validate.py) to check CoNLL-U format compliance, tree well-formedness, and morphosyntactic consistency. The released version passes the official Universal Dependencies validation procedure. The public eval.log generated by the UD release pipeline on 5 May 2026 records both released files, *uz_uzudt-ud-train.conllu* and *uz_uzudt-ud-test.conllu*, as PASSED by validate.py, with the release-validity flag reported as Validity = 1. The same eval.log also reports the UD release-quality rubric, including component scores for lemma coverage, UPOS coverage, morphological-feature coverage, dependency-relation coverage, genre coverage, corpus size, and split size; these rubric scores describe corpus coverage and release quality rather than pass/fail validation errors. The final validated corpus was then partitioned at the sentence level into training and test sets of 483 sentences (5441 tokens) and 198 sentences (2101 tokens), respectively; no separate development split is included in the current public release. The split was stratified at the sentence level across the three source groups represented in the corpus: *Kun shundan boshlanadi, Maqar*, and educational fairy tales from *ertak.uz*. Each source group is represented in both the training and test partitions in approximately its corpus-level proportion, as shown in Table 2. This design preserves domain diversity while supporting reproducible downstream training and evaluation.

## 5 Baseline Benchmark

To provide a benchmark score for future users of UzUDT, we evaluated representative neural NLP pipelines on the UzUDT test set. These baselines give the community an initial reference point for POS tagging, morphological tagging, and dependency parsing on the dataset. The evaluated systems include Stanza with static FastText embeddings [15], Stanza with TahrirchiBERT contextual embeddings, and spaCy with TahrirchiBERT. The results are reported in Table 4.

**Table 4:** Baseline benchmark scores on the UzUDT test set.

System	Representation	UPOS	XPOS	UFeats / Morph Acc	UAS	LAS
Stanza	*FastText cc.uz.300*	79.19	79.81	66.61	69.57	51.24
Stanza	*TahrirchiBERT*	82.45	80.90	65.37	72.05	54.19
spaCy	*TahrirchiBERT*	86.50	86.72	50.55	67.72	45.35

The baseline results indicate that contextual representations from TahrirchiBERT improve both UPOS accuracy and LAS compared with the FastText-based Stanza baseline. Among the reported systems, the Stanza model using TahrirchiBERT achieves the highest LAS, whereas the spaCy[16] model using TahrirchiBERT obtains stronger UPOS and XPOS scores but lower dependency-parsing performance. Overall, these results provide a useful reference point for future studies on Uzbek POS tagging, morphological tagging, and dependency parsing. The machine-readable evaluation outputs and training logs for these baseline experiments are available in the companion parsing repository: https://github.com/SanatbekMatlatipov/robust-parsing-uzbek

## Limitations

The primary limitation of UzUDT is its register focus. The dataset was deliberately constructed from edited literary fiction and educational fairy-tale texts, which makes it a reliable low-noise gold-standard benchmark but not a representative sample of all Uzbek usage. Models trained only on UzUDT are expected to generalize best to other edited Uzbek prose, including literary, pedagogical, and encyclopedic text. Performance may decrease on noisier domains such as social media, where Uzbek frequently appears with Russian code-switching, mixed Latin/Cyrillic spelling, informal transliteration of oʻ/gʻ, abbreviations, emojis, and elliptical utterances. Technical documentation may also introduce named entities, transliterated English terminology, formulaic expressions, and SYM-class tokens that are absent from the present corpus.

To give a limited empirical indication of this expected domain sensitivity, we conducted a controlled robustness probe on the released test split using an UzUDT-trained Stanza pipeline with TahrirchiBERT representations [17]. The probe showed that diacritic removal and light Russian filler-token substitution caused only small changes in parsing scores, while random capitalization produced a moderate decrease and punctuation removal caused the largest degradation because punctuation arcs and clause-boundary cues were removed. These results are used only to clarify the dataset’s expected reuse conditions, not as a full cross-domain evaluation. Values are reproduced from `perturbation_results.json` in the companion review-log repository: https://github.com/SanatbekMatlatipov/robust-parsing-uzbek/tree/main/paper_materials/review_logs

Future releases should therefore extend the Uzbek UD ecosystem with companion treebanks for noisier domains such as social media and technical documentation, while preserving the present dataset’s gold-standard annotation procedure and register coherence.

## Ethics Statement

The authors have read and follow the ethical requirements for publication in Data in Brief and confirm that the current work does not involve human subjects, animal experiments, or any data collected from social media platforms.

## CRediT Author Statement

**Sanatbek Matlatipov:** Conceptualization, methodology, data curation, writing, reviewing and editing, and original draft preparation; **Mersaid Aripov:** Supervision, conceptualization, reviewing, and editing. **Makhmud Bobokandov:** Data curation, original draft preparation; **Gayrat Matlatipov**: Writing-reviewing and editing, data curation, validation, formal analysis.

## Declaration of generative AI and AI-assisted technologies in the manuscript preparation process

During the preparation of this work, the authors partially used Gemini Pro to improve language, readability, academic register, and sentence-level clarity. The sections that received the most significant AI-assisted language polishing were Value of the Data, Data Description, Experimental Design, Materials and Methods, and Limitations. After using this tool/service, the authors reviewed and edited the content as needed and take full responsibility for the content of the published article.

## Data Availability

GitHub)UzUDT (Original data)
